# Bovine Kobuvirus in Calves with Diarrhea, United States

**DOI:** 10.3201/eid2601.191227

**Published:** 2020-01

**Authors:** Leyi Wang, Richard Fredrickson, Michelle Duncan, Jonathan Samuelson, Shih-Hsuan Hsiao

**Affiliations:** University of Illinois, Urbana, Illinois, USA (L. Wang, R. Fredrickson, J. Samuelson, S.-H. Hsiao);; Western Illinois Veterinary Clinic, Quincy, Illinois, USA (M. Duncan)

**Keywords:** Bovine kobuvirus, diarrhea, cattle, viruses, United States

## Abstract

We detected bovine kobuvirus (BKV) in calves with diarrhea in the United States. The strain identified is related genetically to BKVs detected in other countries. Histopathologic findings also confirmed viral infection in 2 BKV cases. Our data show BKV is a potential causative agent for diarrhea in calves.

Bovine kobuvirus (BKV; species *Aichivirus B*, genus *Kobuvirus*, family *Picornaviridae*) was identified initially as a cytopathic contaminant in a culture medium of HeLa cells in Japan in 2003 (*1*). Since then, BKV has been reported in Thailand, Hungary, the Netherlands, Korea, Italy, Brazil, China, and Egypt (*2*–*9*). However, circulation of BKV in North America remains unclear. We report detection of BKV in calves in the United States.

In April 2019, a fecal sample from a 10–14-day-old calf was submitted to University of Illinois Veterinary Diagnostic Laboratory (Urbana, IL, USA) for testing for enteric pathogens. Results of tests for rotavirus, coronavirus, cryptosporidium, and *Escherichia coli* were positive; results for *Salmonella* were negative. 

We extracted nucleic acid from the fecal sample and conducted a sequence-independent single-primer amplification and library preparation by using Nextera XT DNA Library Preparation Kit (Illumina, https://www.illumina.com). We conducted sequencing on a MiSeq (Illumina) using MiSeq Reagent Kit V2 (Illumina) at 500 cycles, as previously described (*10*). We conducted a taxonomic analysis of raw FASTQ files using Kraken version 1 and MiniKraken DB (https://ccb.jhu.edu/software/kraken), which showed 15,582 kobuvirus sequence reads in addition to sequences for *E. coli*, coronavirus, and rotavirus. We assembled the complete genome of BKV IL35164 (GenBank accession no. MN336260) with a genome size of 8,337 nt. We used BLAST (https://blast.ncbi.nlm.nih.gov/Blast.cgi) to search the IL35164 genome and found it is closely related to and shares 89%–91% identities with 4 BKV strains, U-1, EGY-1, SC1, and CHZ. It shares only 77%–82% identity with sheep and ferret kobuviruses.

Sequence analysis showed that IL35164 has a similar genome organization to other BKVs (Figure, panel A) and a 7,392-nt open reading frame encoding 2,463 amino acids, the same length as U-1, EGY-1, and SC1 strains. IL35164 shares polyprotein identities with 4 other BKV strains, 88.5%–90.9% identity in the nucleotide level and 94.9%–96.7% identity in the amino acid level (Appendix Table 1). Comparing individual proteins from 4 other BKVs, IL35164 shared only 80.9%–86.8% nucleotide identity with the leader protein and shares its highest identity, 95.5%–98.8%, with the 3B nucleotide in those strains (Appendix Table 1). In addition to 3B, IL35164 shows higher nucleotide identities, 92.6%–95.4%, to other BKVs in 3D, encoding a viral RNA-dependent RNA polymerase. 

**Figure:**
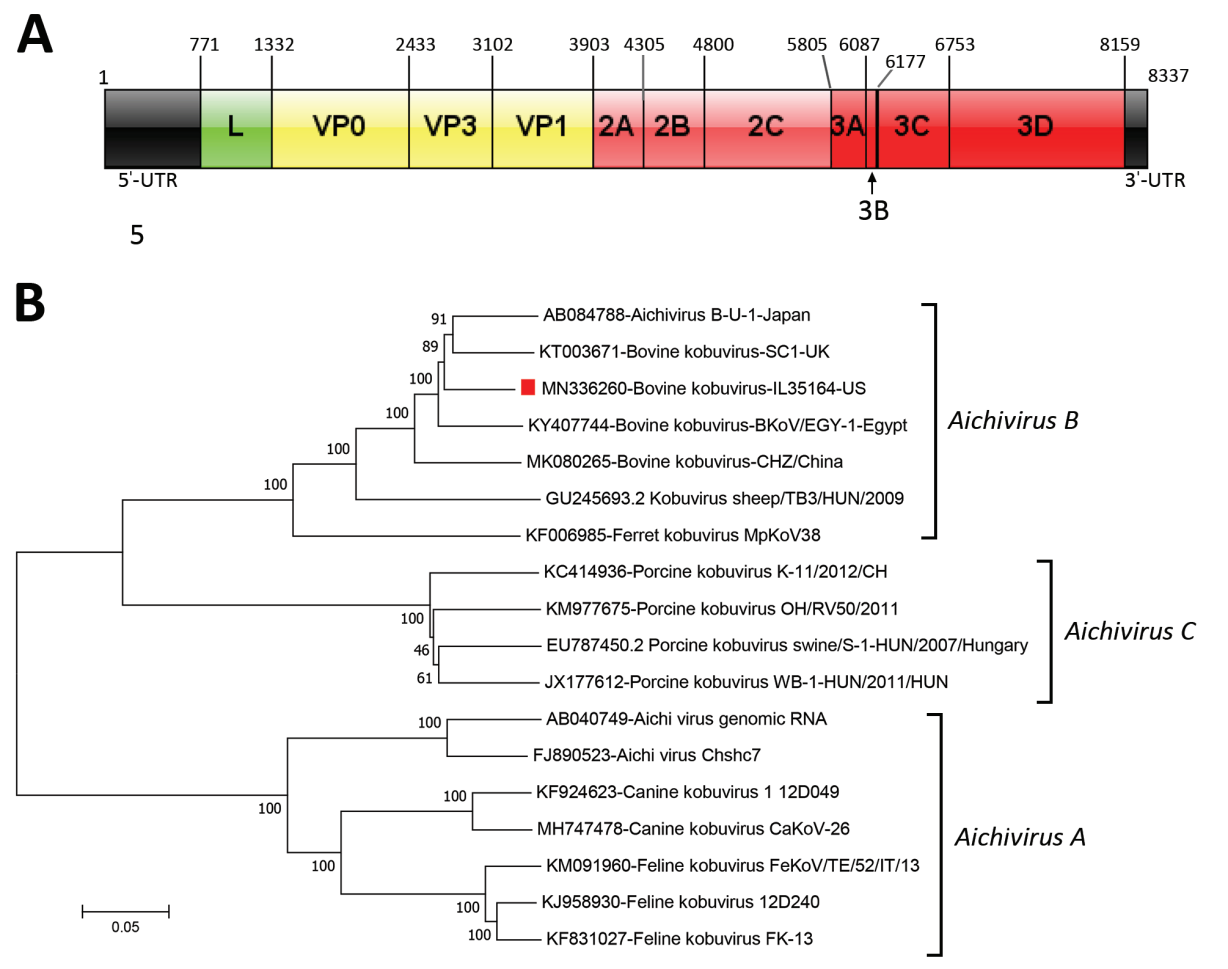
Genome organization and phylogenic tree of bovine kobuvirus IL35164 isolated from cattle, United States. A) Genome organization with each gene’s initial nucleotide position labeled. The 5′ UTR is located in positions 1–770 and the 3′ UTR is located in positions 8160–8337. B) Phylogenetic tree of complete genomes of 3 *Aichivirus* species, *A*, *B*, and *C*. The dendrogram was constructed by using the neighbor-joining method in MEGA version 7.0.26 (http://www.megasoftware.net). Bootstrap resampling of 1,000 replications was performed and bootstrap values are indicated for each node. Red square indicates bovine kobuvirus IL35164 identified in this study. Scale bar indicates nucleotide substitutions per site. UTR, untranslated region; VP, viral protein.

Phylogenetic analysis of the complete genome confirmed that IL35164 correlates with 4 BKVs in the *Aichivirus B* species cluster (Figure, panel B). On the phylogenetic tree of complete VP1 nucleotide sequences, IL35164 clusters with 2 BKV strains from Brazil, BRA1991 and BRA2016, rather than BKVs U-1, EGY-1, SC1, and CHZ (Appendix Figure 1). The relatedness of IL35164 to BRA1991 and BRA2016 in other parts of the genome is unclear because complete genomes of the strains from Brazil are unavailable. IL35164 is related distinctly to U-1, EGY-1, SC1, and CHZ on the phylogenetic tree of partial 3D (Appendix Figure 2).

To further screen BKV in bovine samples, we designed primers and probes (sequences available upon request) targeting 3D to test 9 additional intestinal samples from necropsied calves. Real-time reverse transcription PCR showed 4/9 samples were positive for BKV by cycle thresholds of 23.0 (case IL35146), 29.97 (case IL37122), 32.84 (case IL50179), and 33.61 (case IL34890) but were negative for coronavirus, rotavirus, and bovine viral diarrhea virus (Appendix Table 2). Histopathologic observation of small intestines revealed that 2 cases with diarrhea, IL35146 and IL50179, had necrotizing enteritis with villus atrophy and fusion, suggesting a primary viral infection (Appendix Figure 3). Two other calves without clinically evident diarrhea died, case IL37122 from jejuno-ileal volvulus and case IL34890 from abomasal rupture; both also were positive for BKV.

Among 3 BKV-positive calves with diarrhea, 2 were <1 month of age and 1 was ≈5 months of age. Previous studies reported high prevalence of BKV infection in young calves with diarrhea; 20.9% (38/182) in calves <2 months of age in Brazil and 26.7% (23/86) in calves <1 month of age in South Korea (*5*,*9*). Our study further supports the hypothesis that BKV causes neonatal diarrhea in calves. In addition, BKV also can be detected from cattle without diarrhea or clinical signs of the virus (*1*,*8*).

Since initial identification in 2003 (*1*), BKV has been detected in cattle from several countries, but only from fecal samples; no natural or experimental studies have reported its pathogenesis. Our histologic examination of necropsied cases clearly indicated viral infection, and only BKV was detected, suggesting BKV was the causative agent for diarrhea. Future studies, including virus isolation and virus challenge to calves, are needed to determine whether BKV fulfills the Koch’s postulates as a causative agent for diarrhea in calves.

The prevalence of BKV in the United States remains unknown. Continued surveillance is urgently needed to determine rates and distribution of BKV in North America. Although many partial sequences of 3D and viral protein 1 are available at GenBank, only 4 complete sequences are available, limiting evaluation of BKV. Whole-genome sequencing of both previously and newly discovered BKV isolates is needed to analyze genetic diversity and evolution.

Appendix Additional information on bovine kobuvirus in cattle with diarrhea the United States.
