# Supplementary material for: Bovine Kobuvirus in Calves with Diarrhea, United States
Source: Emerg Infect Dis. 2020 Jan;26(1):176–8. doi: 10.3201/eid2601.191227 (PMC6924891; doi:10.3201/eid2601.191227)
Supplement: Appendix — Additional information on bovine kobuvirus in cattle with diarrhea the United States. [file 19-1227-Techapp-s1.pdf]

Article DOI: https://doi.org/10.3201/eid2601.191227

Bovine Kobuvirus in Calves with Diarrhea, United States

Appendix

Background

Kobuviruses are small, nonenveloped, positive sense, single-strand RNA viruses of the family *Picornaviridae*. *Kobuvirus* consists of 6 known species, *Aichivirus A–F*, according to the latest report of International Committee on Taxonomy of Viruses (https://talk.ictvonline.org/taxonomy). *Aichivirus C–F* are single viral types, but *Aichivirus A* contains 4 types, canine, feline, and murine kobuviruses, and Aichi virus 1. *Aichivirus B* contains 3 types, bovine, ferret, and ovine kobuviruses. The genome size ranges from 8.2 to 8.4 kb. Kobuvirus genome consists of a single open reading frame encoding a large polyprotein cleaved into 3 structural capsid proteins, VP0, VP1, and VP3, and 7 nonstructural proteins 2A, 2B, 2C, 3A, 3B, 3C, and 3D.

Appendix Table 1. Sequence identities of bovine kobuvirus strain IL35164 with other reference strains in different parts of genome*

Virus strain name	L	VP0	VP3	VP1	2A	2B	2C	3A	3B	3C	3D	aa	nt	Complete genome
AB084788-BKV-U-1-Japan	86.8	89.1	89.3	89.3	89.8	91.5	92.0	91.1	97.7	92.3	93.9	95.8	90.9	90.8
KT003671-BKV-SC1-UK	86.4	87.4	91.3	86.2	92.2	92.7	93.2	91.4	98.8	90.2	94.3	96.7	90.8	90.4
KY407744-BKV- EGY-1-Egypt	85.0	86.7	90.8	86.6	92.5	90.9	93.9	91.1	95.5	92.7	95.4	96.7	90.9	90.7
MK080265-BKV-CHZ/China	80.9	84.3	82.8	86.2	91.7	90.3	91.5	90.7	98.8	92.3	92.6	94.9	88.5	83.9
GU245693-sheep/TB3/HUN/2009	67.0	76.9	76.3	76.1	80.8	85.8	87.0	80.6	87.7	85.0	91.4	85.5	81.9	81.4
KF006985-Ferret/MpKoV38	NP	NP	71.5	NP	NP	NP	NP	NP	NP	NP	86.2	81.5	78.6	74.5

*Values represent % identity. aa, amino acid; NP, not performed; nt, nucleotide.

Appendix Table 2. Results of real-time reverse transcription PCR targeting 3D gene of bovine kobuvirus and other viruses and pathology of samples from 5 BKV cases in cattle, United States*

Case no.	Age	Sample type	BKV Ct	Viruses tested, result	Pathology
IL35164	10–14 d	Feces	16.01	Rotavirus, +<br>Coronavirus, +	NA
IL35146	14 d	Intestine	23.00	Coronavirus, –<br>BVDV, –	Villi atrophy and fusion
IL37122	10 d	Intestine	29.97	Coronavirus, –<br>BVDV, –	Jejuno-ileal volvulus with sloughing of villous epithelia
IL50179	21.7 wk	Intestine	32.84	Rotavirus, –<br>Coronavirus, –	Villi atrophy and fusion
IL34890	<30 d	Intestine	33.61	Rotavirus, –<br>Coronavirus, –<br>BVDV, –	Abomasal rupture

*BKV, bovine kobuvirus; BVDV, bovine viral diarrhea virus; C_t, cycle threshold; NA, not applicable;–, negative; +, positive.

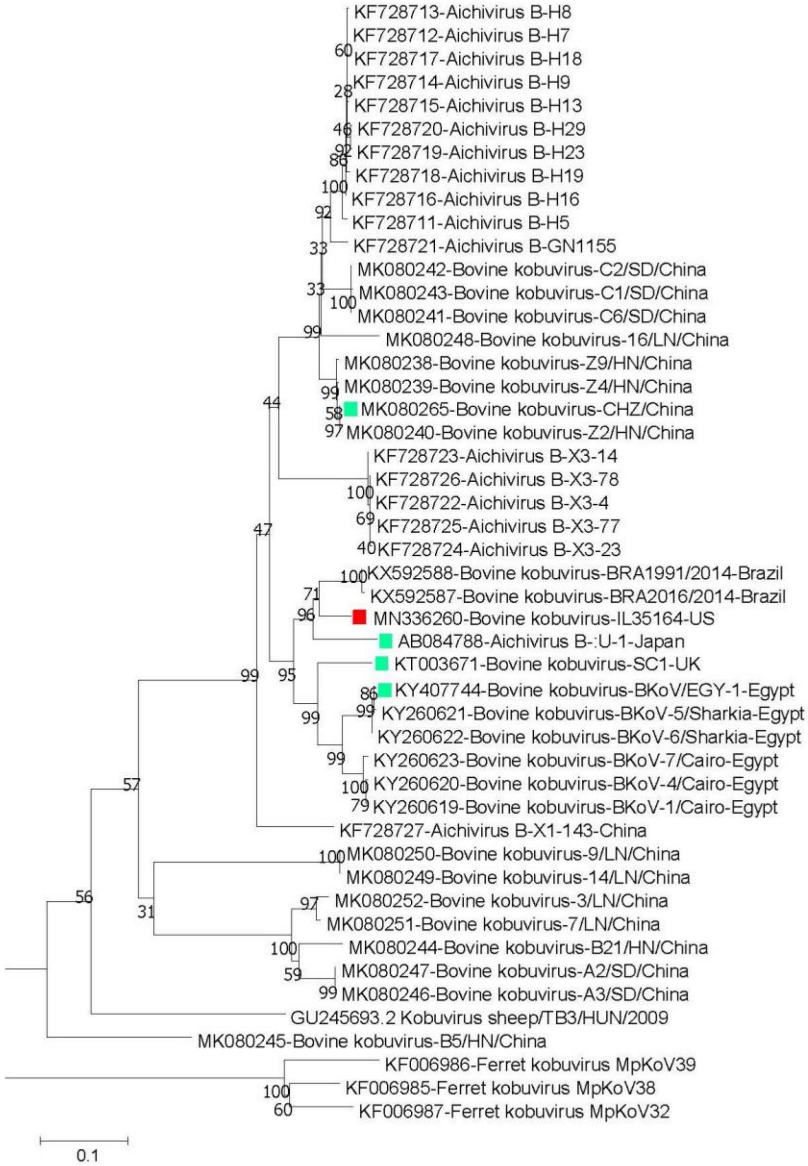

Appendix Figure 1. Phylogenetic tree analysis of VP1 gene of *Aichivirus B* including bovine kobuvirus (BKV) strain IL35164 identified in a calf with diarrhea in the United States. Red square indicates IL35164 from this study; green squares indicate BKV strains with complete genomes, U-1, EGY-1, SC1, CHZ, used for comparison. The dendrogram was constructed by using the neighbor-joining method in MEGA version 7.0.26 (http://www.megasoftware.net).

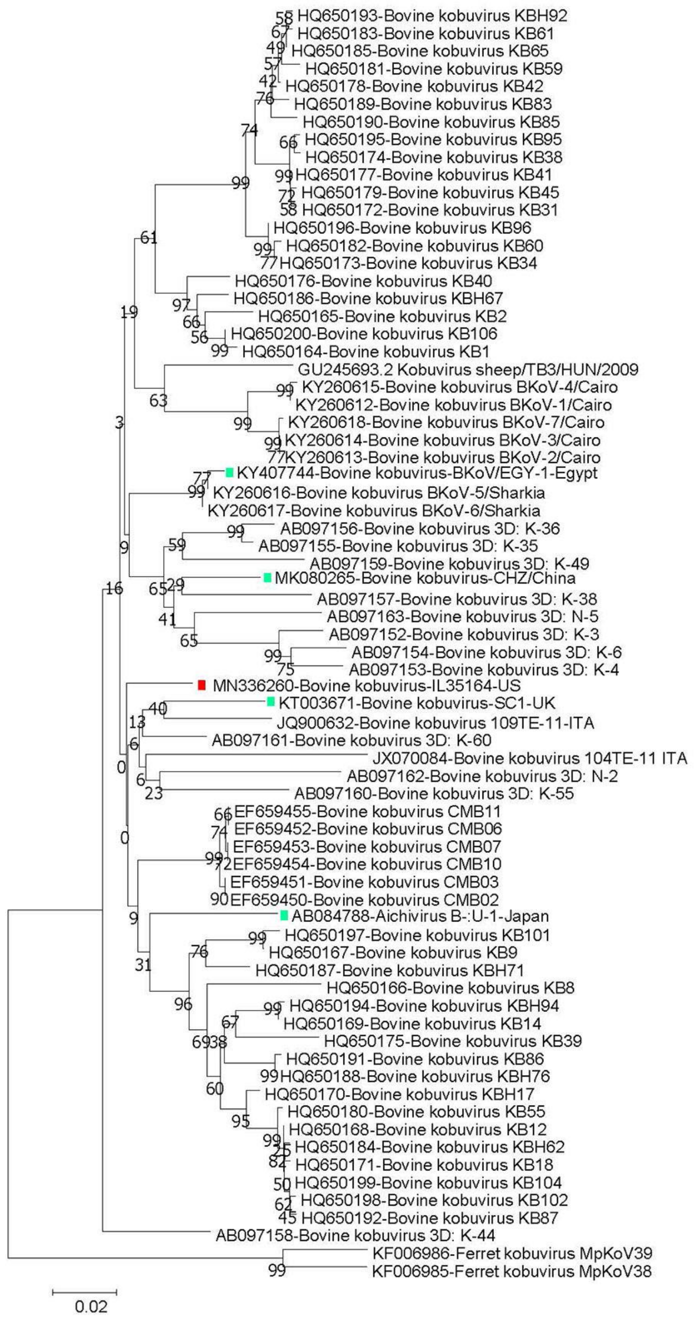

Appendix Figure 2. Phylogenetic tree analysis of partial 3D gene of *Aichivirus B* including bovine kobuvirus (BKV) strain IL35164 identified in a calf with diarrhea in the United States. Red square indicates IL35164 from this study; green squares indicate BKV strains with complete genomes, U-1, EGY-1, SC1, CHZ, used for comparison. The dendrogram was constructed by using the neighbor-joining method in MEGA version 7.0.26 (http://www.megasoftware.net).

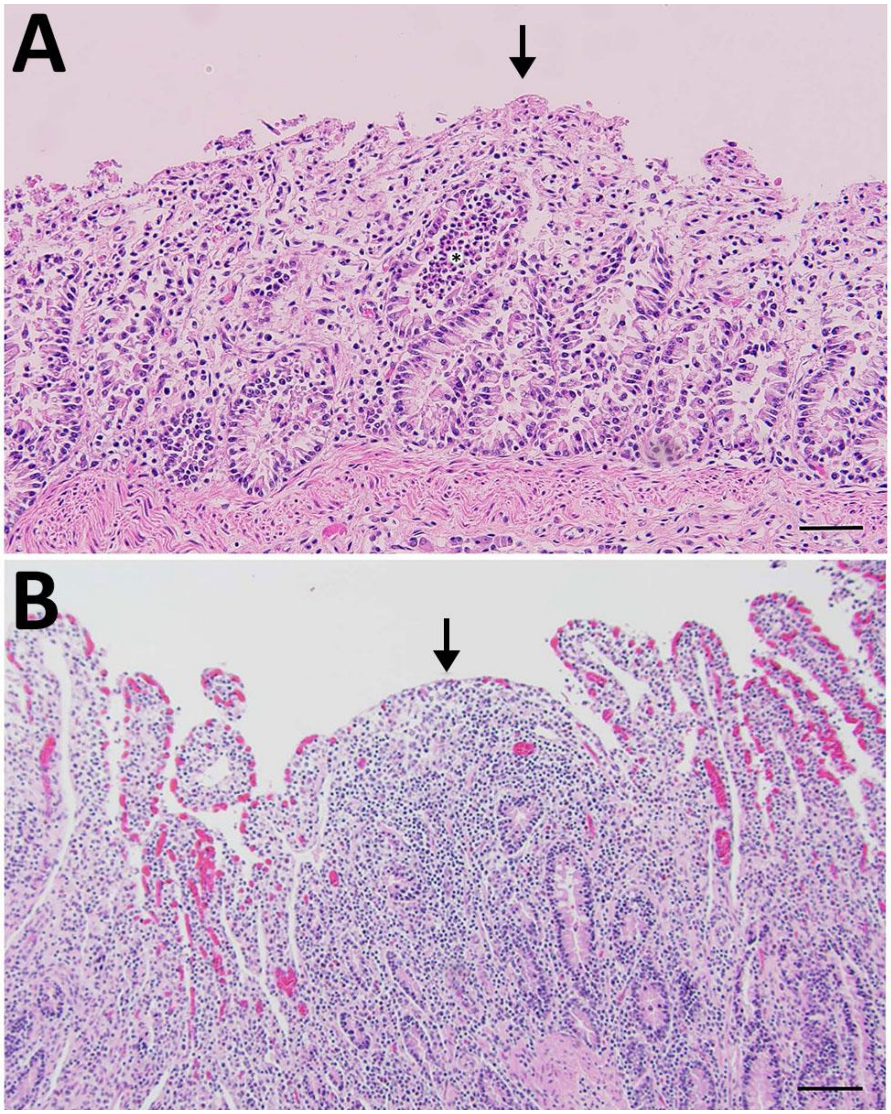

Appendix Figure 3. Photomicrographs of HE stained jejunum sections used for histopathologic analysis of cattle infected with bovine kobuvirus in the United States. A) Case no. IL35146; villous epithelia are largely sloughed, crypts are dilated with clusters of degenerative neutrophils, lamina propria is infiltrated by a small number of lymphocytes, plasma cells, and neutrophils. Arrow indicates villi of the jejunum, which are short, blunted and fused; asterisk indicates crypt abscess. B) Case no. IL50179; villous epithelia are largely sloughed, lamina propria is infiltrated by lymphocytes, plasma cells, and eosinophils, and moderate villous lymphangiectasia is visible. Arrow indicates jejunal villi, which are fused and atrophied with club-shaped tips. Scale bar indicates 100 µm.
